# Right Paraduodenal Hernia with Midgut Malrotation

**DOI:** 10.5334/jbsr.1876

**Published:** 2019-10-09

**Authors:** Joon Ho Cho, Seung Soo Kim, Woong Hee Lee

**Affiliations:** 1 Department of Radiology, Soonchunhyang University College of Medicine, Cheonan Hospital, Cheonan-si, KR

**Keywords:** Hernia, Congenital abnormalities, Computed tomography

## Abstract

**Main teaching point:** Right paraduodenal hernia typically occurs in the fossa of Waldeyer, with the herniated bowel sac located posterior to the right colic artery and vein.

## Case History

**Figure 1:**
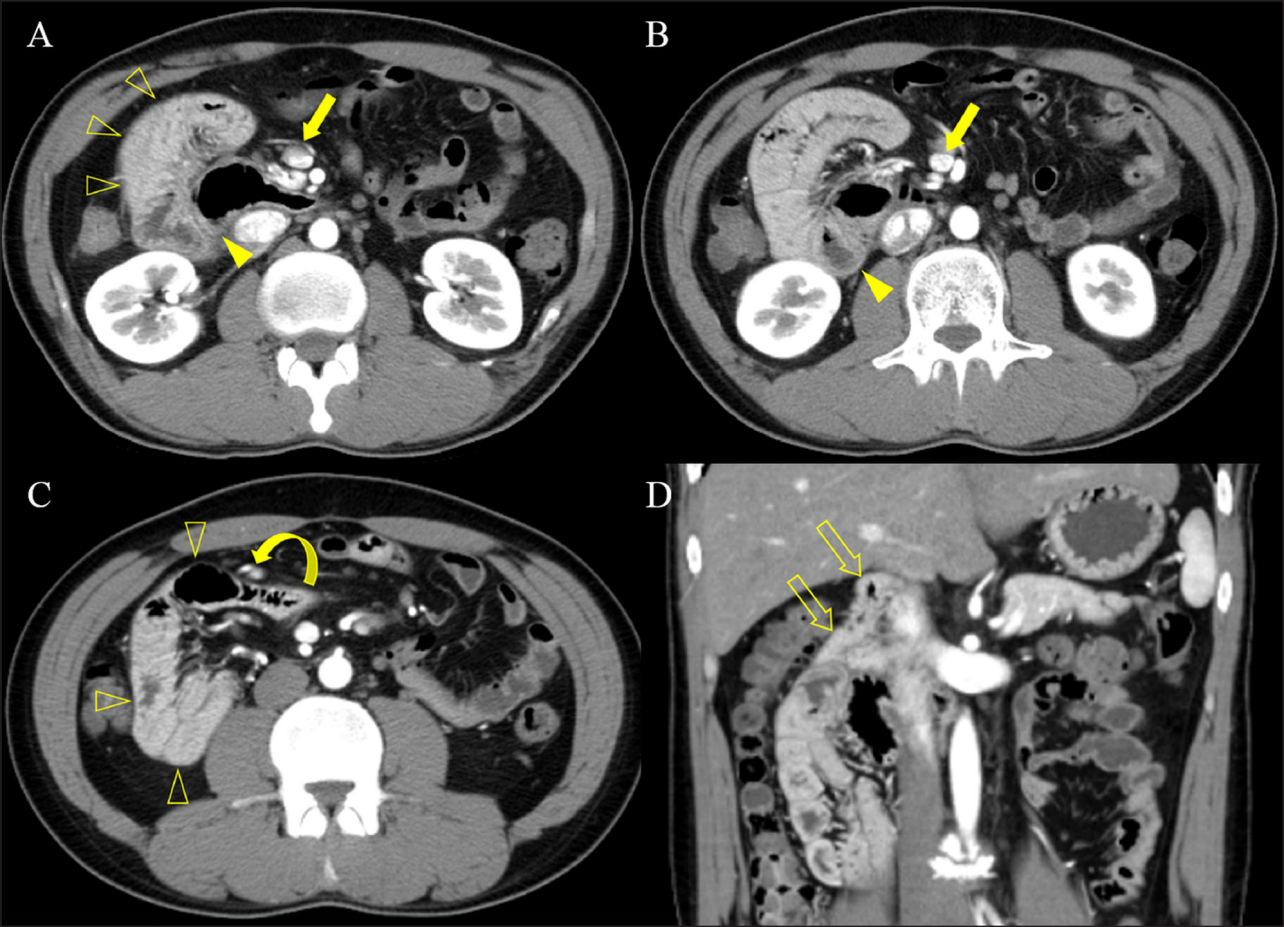


A 30-year-old man was admitted with a one-year history of intermittent abdominal pain and a more recent four-month history of projectile vomiting. Laboratory tests were normal. Contrast-enhanced computed tomography (CT) (Figure 1A–C) identified an unusual cluster of saclike pseudo-encapsulated small bowel loops (open arrowheads) in the right side of the abdomen. The proximal jejunum (arrowhead) was located posterolateral to the superior mesenteric vein (arrow). The right colic artery and vein (curved arrow) were displaced anteriorly by the entrapped cluster of bowel loop. Coronal reformatted CT image (Figure 1D) demonstrated that the duodenum (open arrows) that did not cross the midline. The patient underwent surgery and was diagnosed having right paraduodenal hernia (RPH) and midgut malrotation.

## Comment

RPH is a type of internal hernia and is frequently associated with intestinal malrotation. Although RPH is relatively rare compared with left paraduodenal hernia, it causes high mortality as high as 50%. RPH occurs in the fossa of Waldeyer, which is typically located inferior to the third portion of the duodenum and behind the small bowel mesenteric root. During the 6th–10th week of embryonic period, midgut normally rotates 270° counterclockwise manner. Then, the prearterial jejunum lies in the left upper quadrant and the postarterial jejunum is located in the right lower quadrant. The fossa of Waldeyer is thought to result from incomplete rotation of the prearterial jejunum, and subsequent failure of fusion between the mesentery and duodenal third portion [1].

CT is the standard of reference for the diagnosis of internal hernia and its complications. In cases of internal hernia into a fossa in the retroperitoneum, the bowel loop usually appears as a pseudo-encapsulated sac-like structure on CT. This appearance is one of the clues for diagnosing an internal hernia. When a RPH occurs, the right colic artery and vein are located anterior to the herniated bowel sac. Internal hernias, including RPH, can lead to a closed loop obstruction, subsequent intestinal ischemia and strangulation. Decreased enhancement of the bowel wall is an important CT finding suggesting necrosis [1]. Normally, the duodeno-jejunal junction lies to the left of the spine at the level of duodenal bulb on CT. However, in patients with midgut malrotation, the duodenum does not cross the midline and the duodeno-jejunal junction is located in the right side of the abdomen [1]. Patients with RPH need surgery in order to prevent future bowel strangulation.
